# HIV Self-Testing Uptake and Intervention Strategies Among Men in Sub-Saharan Africa: A Systematic Review

**DOI:** 10.3389/fpubh.2021.594298

**Published:** 2021-02-19

**Authors:** Akeen Hamilton, Noah Thompson, Augustine T. Choko, Mbuzeleni Hlongwa, Pauline Jolly, Jeffrey E. Korte, Donaldson F. Conserve

**Affiliations:** ^1^Department of Health Promotion, Education and Behavior, University of South Carolina, Columbia, SC, United States; ^2^Department of Biological Sciences, University of South Carolina, Columbia, SC, United States; ^3^Faculty of Epidemiology and Population Health, London School of Hygiene & Tropical Medicine, London, United Kingdom; ^4^Discipline of Public Health, School of Nursing and Public Health, University of KwaZulu-Natal, Durban, South Africa; ^5^Department of Epidemiology, University of Alabama at Birmingham, Birmingham, AL, United States; ^6^Department of Public Health Sciences, Medical University of South Carolina, Charleston, SC, United States; ^7^Department of Prevention and Community Health, The George Washington University, Washington, DC, United States

**Keywords:** HIV, self-testing, men, Sub-Sahara Africa, systematic (literature) review

## Abstract

**Background:** HIV testing is an essential gateway to HIV prevention and treatment services. However, HIV testing uptake remains low among men due to stigma, discrimination, and confidentiality concerns. HIV self-testing (HIVST) is an alternative HIV testing method that can address many of these barriers for men. We conducted a systematic review to examine HIVST uptake and intervention strategies among Men in Sub-Saharan Africa.

**Methods:** We used a systematic approach to survey literature published from January 2010 to June 2020 using five electronic databases (PubMed-Medline, CINAHL Complete, PsychINFO, Google Scholar, and Web of Science) and a manual search. Studies were included if they were peer-reviewed, published in English, and examined HIVST willingness, uptake, and/or linkage to care and included men in Sub-Saharan Africa.

**Results:** Sixty-three articles related to HIVST were reviewed. Of the included articles, 37 discussed HIVST uptake/acceptability and 24 discussed intervention strategies. Both oral swab and finger-prick methods had high acceptability with ease of access and availability of the test cited as important by men. Free HIVST kits were preferred by men. Secondary distribution of kits via peers, sexual partners, and female sex workers were successful.

**Conclusion:** HIV self-testing is highly acceptable to men. More efforts are needed to develop policies to implement HIVST programs targeting men in Sub-Saharan Africa, including a focus on linkage to care in sub-Saharan Africa. Future interventions should directly target men independently in tandem with using peers and their romantic partners to promote self-testing among men in sub-Saharan Africa. HIVST kit distribution strategies should be combined with services that can offer confirmatory tests and counseling for men as well as linkage to care.

## Introduction

As of 2018, almost 37.9 million people worldwide were infected with HIV (1), with only 75% of people living with HIV (PLWH) globally being aware of their HIV status (1). The Joint United Nations Programme on HIV/AIDS (UNAIDS) responded by developing an ambitious treatment plan to end the HIV/AIDS epidemic. The objective was for 90% of all PLWH to become aware of their HIV status, 90% of those be linked to sustained antiretroviral treatment (ART) so that 90% of people receiving ART could achieve viral suppression (2). HIV testing is an essential gateway to initiate HIV prevention and treatment services. Yet, most individuals who are at high risk of contracting HIV or who are already infected with HIV are not accessing HIV testing at a high enough rate due to fear of stigmatization, inadequate treatment by healthcare workers, and/or confidentiality concerns (2). In sub-Saharan Africa, the increasingly widespread availability of HIV testing remains hindered by the perceived psychological burden of having to live with HIV, financial barriers, as well as gender inequality (3). Furthermore, men in sub-Saharan Africa are less likely to be self-aware of their HIV status compared to their female counterparts (3). This can be attributed to the exposure of women to HIV testing through antenatal services as well as to misguided masculinity norms (4, 5). In nations like Uganda and Tanzania, a lack of knowledge of HIV status is the limiting factor in getting people to engage in prevention and treatment programs (6). The 2016–2017 Tanzania HIV Impact Survey showed that only 45% of men living with HIV (MLWH) were aware of their positive HIV status (7). Eighty-six percent of MLWH who knew their HIV status reported initiation of ART, and 84% of those undergoing ART had been virally suppressed and were significantly less likely to transmit HIV to others (6). HIV screening is a hallmark in being able to provide linkage to care and in turn halt the transmission of HIV. Various interventions have been employed throughout the last decade to determine the most effective way of encouraging men to get tested for HIV, including antenatal clinic-based testing for upcoming fathers, community-based testing, workplace testing, home-based testing, and most promising of all, self-testing.

HIV self-testing (HIVST) is an alternative HIV testing method that can overcome many barriers to testing, including stigma, privacy concerns, time and expense associated with traveling and waiting at the clinic for men. In HIVST an individual can use a kit to collect a specimen, perform the test (usually a rapid diagnostic test) which screens for HIV-1/2 antibodies or the HIV-1 p24 antigen (8), and interpret the test results for themselves. A positive result requires confirmatory testing at a clinical facility which allows for more accurate diagnoses as well as for those persons to easily become linked to ART (8). This promising approach overcomes the initial stigmatization of HIV testing by promoting privacy and security (8). In 2018, the World Health Organization (WHO) reported that 59 countries worldwide had taken up HIVST and 53 additionally were developing policies (8). However, around two thirds of these nations have upper middle- or high-income status, including Australia, Brazil, France, Moldova, the UK, and USA (8). HIVST pricing in low- to middle-income countries, like those in sub-Saharan Africa, is expected to decrease significantly thanks to the Bill & Melinda Gates Foundation's recent agreement to support the affordable sale of Oraquick Self-Testing Kits in order to continue the scale-up of HIV testing in these higher risk areas (9). In November 2018, the UNITAID Self-Testing Africa Initiative distributed nearly 2.3 million HIVST kits in East and South Africa, with a significant number given to countries like South Africa, Zimbabwe, Malawi, Zambia, eSwatini, and Lesotho (9). Between 2015 and 2017, nearly 628,700 self-test kits were distributed to Malawi, Zambia, and Zimbabwe, with close to half of all people self-testing being men and between 14 and 27% not having previously tested for HIV (9). One study in Kisumu, Kenya that distributed self-test kits to women receiving antenatal services for their male partners, discovered that 90.8% of male partners had tested for HIV in the self-testing group compared to only 51.7% of partners in the facility-based testing group (10). Another study performed by PopART in Zambia that observed the rate of uptake when HIVST were distributed door-to-door showed success in increasing awareness of HIV status among men (11).

We conducted a systematic review to examine the HIVST literature focusing on men in Africa. While past research has presented findings from a global review regarding HIVST (12–17), there are currently no systematic reviews published for HIVST uptake and intervention strategies among men in sub-Saharan Africa. In this review, we aim to systematically identify relevant articles to address this gap and to provide implications for future research.

## Methods

This review adopted and followed the guidance provided by the Preferred Reporting Items for Systematic Reviews and Meta Analyses (PRISMA) (18) and was registered with PROSPERO (registration number: CRD42020138729). A literature search was completed by a trained librarian for articles which matched the criteria for inclusion. To ensure ample coverage in the search process, the following electronic databases were surveyed: CINAHL, PsycINFO, PubMed, Web of Science, and Google Scholar. Both Boolean-paired keywords and controlled vocabulary pertaining to HIVST strategies for men in sub-Saharan Africa were used. Search strategies included terms, such as HIV, HIV self-testing, HIVST, HIV testing, Self-Testing, Men, Male(s), Willingness, Uptake, Intervention(s), Africa, Sub-Saharan Africa, West-Africa, East Africa, southern Africa, and all sub-Saharan African country names.

### Inclusion Criteria

Articles were included in this review if they met the following criteria: (1) the research was conducted in sub-Saharan Africa, (2) reported findings on HIVST, among men aged 16 years or older, (3) the research was peer-reviewed and published in English. Articles were excluded if the research was unpublished, if they were written in languages other than English, or if they were not published between January 2010 and June 2020. Articles were considered to be “current literature” if they were published within the past 10 years; therefore, articles published prior to this time were not included in this review. The included articles focused on men who were deemed as being at-risk for HIV infection and living in sub-Saharan Africa.

### Data Extraction

Two research team members independently reviewed the results of the database search in an Endnote file. The team members first reviewed the titles and abstracts of all articles, after duplicate articles were removed, in order to assess the relevance of each article. The articles were grouped into one of two categories, either “Selected for Full-Text Review” or “Does Not Meet Inclusion Criteria.” Data were then extracted from the articles categorized as selected for full-text review. Ninety-seven articles were selected to be reviewed in full. The two research members summarized the selected articles according to their methods and findings with the aim of assessing if they met the full inclusion criteria. The research members also independently read the included articles in their entirety and summarized their methods, design, and results in order to confirm the appropriateness for being included in the final sample of included articles. Any discrepancies or confusion pertaining to the included articles for which consensus could not be reached by the two research members, was settled by a third research team member.

### Quality Assessment

In order to assess the methodological and research quality of each article that is included in this review, appropriate quality assessment tools were used. Two team members independently rated each included article using a pre-determined and agreed upon acceptable scoring requirement for each assessment. The Critical Appraisal Skills Programme (CASP) (19) was used to evaluate qualitative studies; included articles had to meet at least seven of ten listed criteria. Included articles evaluated using the Quality Assessment Tool for observational cohort and cross-sectional studies (20) were required to meet at least eleven of fourteen listed criteria. Included articles evaluated using the Cochrane Risk of Bias Tool for randomized trials (21) were required to have a low level of assessed bias in the four domains (selection, performance, attrition, and other). Studies that scored poorly on either of the quality assessment tools were removed from inclusion.

### Chosen Methodology

A narrative synthesis approach was used to present the results of this review. Narrative synthesis allows for the synthesis of the findings of multiple studies in a qualitative manner (22). We organized each article by themes, such as HIVST uptake and intervention strategies. The findings of each article were matched with their appropriate theme; however, some articles were included in multiple themes.

## Results

**Figure 1:**
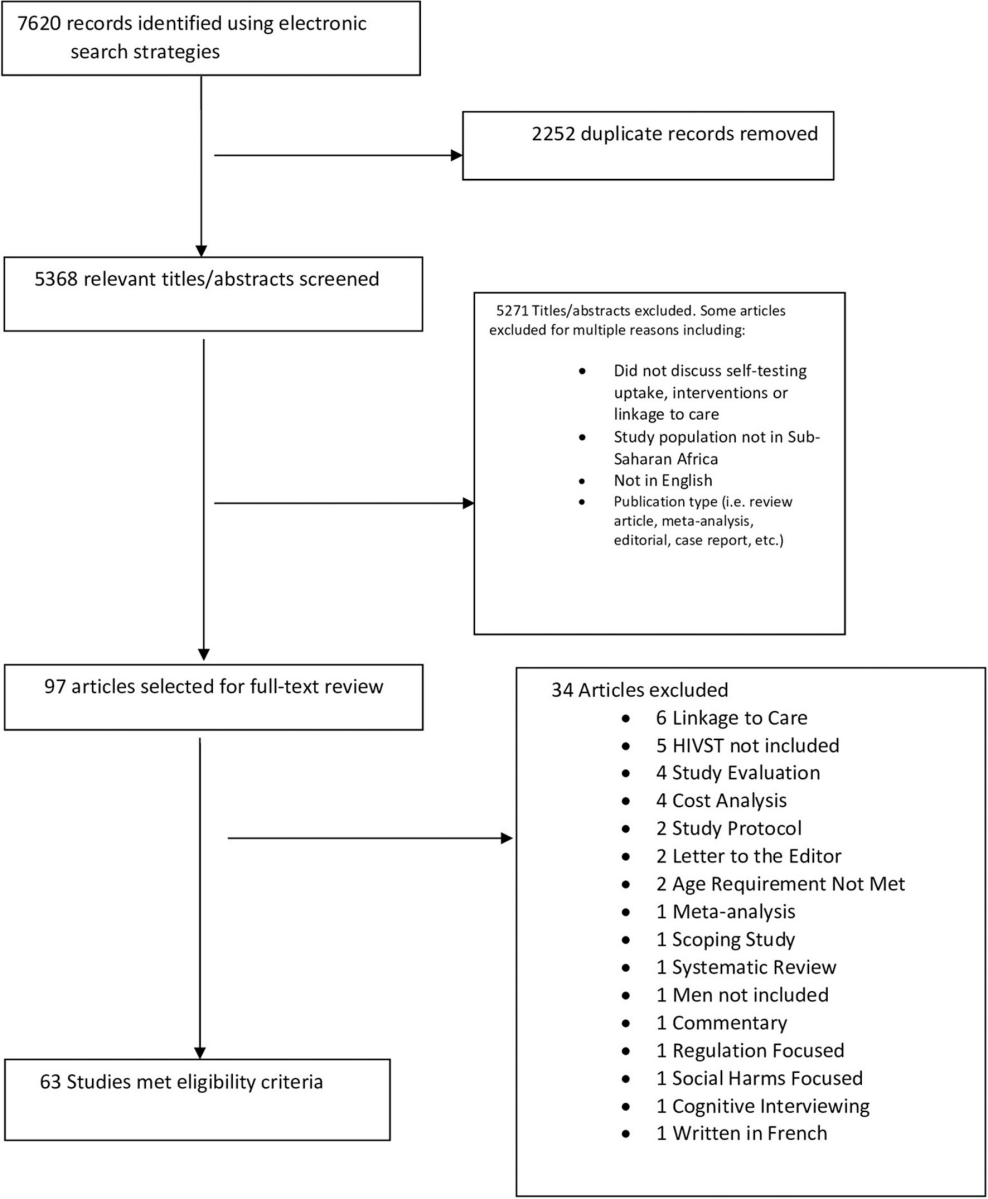
PRISMA flow diagram for article inclusion/exclusion.

The results of the database searches yielded 7,620 articles; following the removal of duplicate articles, 5,368 articles remained (Figure 1). Articles (*n* = 5,271) were then excluded for multiple reasons, such as not discussing HIVST, study populations not being in sub-Saharan Africa, article not being written in English, or the article was not a peer-reviewed original article. Ninety-seven articles were selected for full-text review. Thirty-six articles were excluded because they did not meet the full-inclusion criteria. Sixty-one articles met the full-inclusion criteria and are presented in this review (see Figure 1). Fourteen sub-Saharan countries were represented in included studies (Table 1). The frequency of represented countries in included articles were as follows: Botswana (1), Ethiopia (1), Kenya (14), Lesotho (1), Malawi (12), Mozambique (1), Nigeria (1), Rwanda (2), Senegal (1), South Africa (21), Tanzania (4), Uganda (9), Zambia (5), and Zimbabwe (4). The designs of the articles included: cluster randomized trial (2), cohort study (4), cross-sectional survey (7), demonstration study (4), discrete choice experiment (3), experimental exploratory design (1), feasibility study (1), implementation project (1), individual-based scholastic model (1), longitudinal study (1), mixed methods (1), multiple models of distribution (e.g., community based, mobile outreach, workplace, public health facilities, etc.) (1), non-experimental descriptive study (1), prospective study (2), qualitative (e.g., in-depth interviews, focus groups, etc.) (26), randomized clustering (1), randomized controlled trial (RCT) (9), single-arm pilot trial (1), and three-phase trial (1). Of the included articles, 37 focused on HIVST uptake/acceptability, and 24 focused on intervention strategies; however, data extracted from each article is presented under its appropriate subsection regardless of study focus.

**Table 1:** Included articles.

**Theme**	**References**	**Included countries**	**Study population**	**Study design**	**Sample size**	**Main findings**
HIVST uptake/Acceptability	Burke et al. (23)	Uganda	Healthcare providers and community members in high-risk fishing communities	In-depth interviews and focus groups	30 men; 25 women	Most participants were not familiar HIVST but believed there were benefits associated: privacy, convenience, and being able to test before sex. Perceived barriers included absence of professional support, poor disposal of kits, and delayed linkage to care.
	Cambiano et al. (24)	Botswana, Lesotho, Malawi, Nigeria, Rwanda, Tanzania, Uganda, Zambia, Zimbabwe	Women having transactional sex, young people, adult men	Individual-based scholastic model	Not specified	Community-based HIVST had the greatest impact with adult men with an average of 1,500 HIV infections averted.
	Choko et al. (25)	Malawi	Men and women	Formative qualitative study	8,643 men; 8,017 women	76.5% of residents self-tested during months a 12-month period. Persons aged 16–19 were most likely to test.
	Choko et al. (26)	Malawi	Pregnant women and their male partners	Formative qualitative study	18 men; 20 women	Male partners reported a preference for HIVST due to its perceived privacy and reduction of associated stigma.
	Conserve et al. (27)	Tanzania	Men	In-depth interviews	23 participants	Seventy-eight percent of participants had never heard of HIVST; sixty-five percent of participants were willing to use HIVST in the future.
	Conserve et al. (28)	Tanzania	Men	In-depth interviews	23 men	HIVST willingness was highly acceptable among both male ever-testers and never-testers. Some 72% of ever-testers vs. 67% of never-testers reported being willing to self-test.
	Dzinamarira et al. (29)	Rwanda	Key stakeholders	In-depth interviews	10 men; 3 women	Key stakeholders perceived HIVST as an effective initiative that may be used to increase uptake of testing services for underserved populations in Rwanda.
	Gumede and Sibiya (30)	South Africa	Men and women	Quantitative, non-experimental descriptive study	442 healthcare users	Most healthcare users (HCU) (69.9%), consisting of both men and women, reported having heard of HIVST in South Africa. Most HCU (81.2%) perceived HIVST as a strategy that could lead to more people knowing their HIV status.
	Harichund et al. (31)	South Africa	Men and women	Qualitative comparative cross-over	12 men; 28 women	Naïve testers were confident in performing unsupervised HIST but reported desiring more counseling support during the testing process.
	Harichund et al. (32)	South Africa	Men and women	Qualitative comparative cross-over	12 men; 28 women	Men deemed HIVST acceptable because of its convenience and efficiency.
	Harichund et al. (33)	South Africa	Men and women	Focus groups and individual interviews	63 participants	HIVST is advantageous when provided in combination with existing services. All distribution models had high male participation in the country.
	Hatzold et al. (34)	Malawi, Zambia, Zimbabwe	Adults and adolescents	Multiple models of distribution (e.g., community based, mobile outreach, workplace, public health facilities, etc.)	294,508 men; 130,223 women	Male partners believed secondary distribution of HIVST kits to be acceptable due to its convenience, confidentiality, privacy, and its ability to allowed men to avoid the clinic
	Hector et al. (35)	Mozambique	Adolescents	Demonstration study	496 students	Over 80% of participants selected directly assisted HIVST compared to standard FS testing and of those who selected HIVST, 20% opted to perform HIVST at home. More than three-fourths of participants (76%) preferred to do HIVST at the health center due to the presence of a counselor.
	Hershow et al. (36)	Malawi and Zambia	Male partners; pregnant and postpartum women	Qualitative formative study	28 male partners; 80 pregnant/	Of three male partner HIV testing strategies (HIV partner notification, partner HIV self-testing, and partner home-based HIV testing) the majority of participants (both men and women) accepted all three partner testing modalities; however, male
					post-partum women	partners were split in their preferences for the three partner testing modalities. Most women and male partners thought home-based testing and secondary distribution of HIV self-test kits were acceptable. Secondary distribution of HIVST kits was thought to be convenient, ensured confidentiality, allowed men to avoid the clinic, and allows for couples testing privately. Home-based testing was thought to be convenient and would provide savings in time and transport money, and helpful to have health workers present to provide counseling.
	Janssen et al. (37)	South Africa	Men and women	Observational cohort study	14 men; 16 women	A smartphone app used in tandem with an oral HIVST was able to help people through the self-testing process by providing counseling and care and simplifying the process of self-testing. The app was able to multiple common HIV testing barriers, such as lack of confidentiality, wait times and testing locations. The app also enabled testing services outside a clinic context or within a clinic; however, an additional layer of privacy was added by using the app. Participants were able to use the app-based HIVST strategy unsupervised at home, unsupervised alone at the Kiosk around the clinic, or supervised under direct supervision of staff at the clinic.
	Kebede et al. (38)	Ethiopia	HCWs	Cross-sectional study design triangulated with qualitative method	307 HCWs	Both oral swab and finger-prick methods had high acceptability. Ease of access and the availability of the test were cited as being of importance.
	Knight et al. (39)	South Africa	Men and women	In-depth interviews	50 lay users	Individual motivations for HIVST included perceived benefits of access to treatment. HIVST was regarded as convenient, confidential, reassuring and an enabling new way to test with one's partner.
	Kumwenda et al. (40)	Malawi	Cohabitating couples	Analysis of baseline data within a 12-month qualitative longitudinal cohort study nested into a cluster randomized trial	17 couples (34 participants)	Men sometimes required persuasion even though they believe HIVST is more flexible than traditional testing.
	Kurth et al. (41)	Kenya	Men and women	Prospective validation study	161 men; 78 women	The acceptability rate for HIVST was 94%. The main theme in the behavioral study was affordability; participants were willing to pay up to 111 Ksh (around $1.25 USD) for an HIVST kit.
	Lebina et al. (42)	Uganda	Men and women	HIV self-screening demonstration project	808 men; 809 women	Some 68.7% of participants selected unsupervised HIVST while 25% opted for supervised HIVST and 6.3% chose semi-supervised.
	Lyons et al. (43)	Senegal	Men and women	Experimental design	1,959 participants	Most participants (74.5%) were comfortable using HIVST, 86.1% found the instructions easy to follow, and 94.4% believed their family or friends would use it.
	Majam et al. (44)	South Africa	Lay users	Cross-sectional study	777 men; 633 women	Participants had a high average usability index of 93.8% for HIVST; some 96.6% of participants found HIVSTs easy to use.
	Makusha et al. (45)	South Africa	Key stakeholders	In-depth interviews	12 participants	Stakeholders expressed high enthusiasm regarding HIVST, its scale-up, and the development of HIVST policies and programming. Perceived barriers included a lack of counseling and Difficulty in ensuring linkages to care among those with positive results.
	Martínez-Pérez et al. (46)	South Africa	Men and women	Mixed-methods research	9 men; 11 women	Participants believed that home O-HIVST uptake would not necessarily lead to higher uptake. It was also believed that men that would show the most interest in using home O-HIVST compared to their female counterparts.
	Martínez-Pérez et al. (47)	South Africa	Men and women	Cross-sectional study	741 men; 1,457 women	Only 3.9% of men had heard about oral HIVST prior to the study. Uptake of oral HIVST was 25.4%
	Matovu et al. (48)	Uganda	Pregnant women and their male partners	Cross-sectional qualitative study	62 FGD participants with pregnant women and 30 IDI with male partners of pregnant women	Most women were willing to take the kits to their male partners and male partners reported being willing to use HIVST kits provided to them by their female partner. Women believed that HIVST could help to improve couples' HIV testing.
	Matovu et al. (49)	Uganda	Pregnant women and their male partners	In-depth interviews	32 participants	Men reported skepticism regarding HIVST and whether or not the kits could actually test for HIV, but this was not a deterrent to its use. Both men and women believed HIVST is a strategy that could address men's lack of time to go to the health facilities to test for HIV.
	Mokgatle and Madiba (50)	South Africa	Technical vocational education and training college students	Cross-sectional survey	1,565 male and 2,040 female students recruited from 13 colleges	Less than half of students (46.2%) were knowledgeable of what HIVST is prior to the administration of the survey. Still, HIVST acceptability was high among the students (87.1%); three-quarters of students were willing to purchase an HIVST kit and many reported being willing to self-test with their partners.
	Njau et al. (51)	Tanzania	Individuals, community leaders, experts	Focus groups and in-depth interviews	21 men; 33 women	Participants reported positive attitudes toward HIVST, supportive perceived norms, and self-efficacy.
	Peck et al. (52)	Kenya, Malawi, South Africa	Lay users	Formative usability research—In-depth interviews	150 Participants	Users found instructions for HIVST to be confusing and/or difficult to follow. Less than 25% of participants completed the test successfully without errors. Results interpretation was difficult for participants.
	Ritchwood et al. (53)	South Africa	Young adults	Focus groups and direct observation	19 men; 16 women	Participants deemed HIVST acceptable due to its privacy, ease of use, and trustworthiness.
	Sibanda et al. (54)	Zimbabwe	Men and women	Discrete choice experiment	128 men; 168 women	The strongest preference for kits was price—every $1 increase in price increased disutility. Door-to-door delivery of kits was highly preferred compared to kit distribution to batch deliveries.
	Spyrelis et al. (55)	South Africa	Men and women	Focus group discussions	118 participants	HIVST was deemed acceptable; however, men had concerns (potential suicidality) regarding the lack of HIV counseling associated with HIVST. Privacy and confidentiality were perceived benefits of HVST.
	van Dyk (56)	South Africa	Men and women	Semi-structured questionnaire	147 men; 319 women	Preferences of testing were associated with patient autonomy, violation of human rights, confidentiality and privacy, fear of discrimination and stigma, and an aversion to mandatory face-to-face counseling.
	van Dyk (57)	South Africa	Men and women	Semi-structured questionnaire	147 men; 319 women	Twenty-two percent of participants preferred HIVST; however, 66% of participants (mostly men) preferred client-initiated testing. Participants reported being willing to use HIVST if it included telephone counseling and if it were available in their communities.
	van Rooyen et al. (58)	Kenya, Malawi, South Africa	Government policy makers, academics, activists, donors, procurement specialists, laboratory practitioners, and health providers	In-depth interviews	54 participants	Participants were in support of the idea of an accurate, easy-to-use, rapid HIVST and believed that this could increase testing across all populations.
	Zanoli et al. (59)	Zambia	Households	Structured survey questionnaire	1,617 Participants	After being informed about HIVST, 91% of participants reported being comfortable with using a self-test; 87% believed that HIVST would increase their likelihood of testing.
Intervention strategies	Asiimwe et al. (60)	Uganda	Men and women	Un-blinded randomized non-inferiority trial	141 men; 105 women	Participants were randomized to either an unsupervised HIVST group or a provider supervised HIVST group. Unsupervised HIVST was able to identify 90% of HIV-infected persons.
	Chang et al. (61)	Zimbabwe	Men and women	Randomized clinical trial	1,155 men; 2,841 women	Participants were provided vouchers to be redeemed for HIVST within 1 month at prices between $0 and $3 at multiple sites. A high sensitivity to price for HIVST was realized among men, rural residents, and persons who had never tested for HIV. Reduced-priced or free tests increased demand
	Choko et al. (25)	Malawi	Men and women	Prospective study nested within a cluster-randomized trial	6,124 men; 7,868 women	Participants received pre-test counseling, instructions on how to perform HIVST, and were asked to demonstrate their understanding of how to use the kit; 10% of participants required help or made errors while using the kits. The estimated uptake of HIVST was >80%. Uptake was greater among women than men.
	Choko et al. (62)	Malawi	Adult members of 60 households and 72 members of community peer groups	Population-weighted randomized clustering	298 adult participants	Participants were offered self-testing plus confirmatory HTC (parallel testing with two rapid finger-prick blood tests), standard HTC alone, or no testing. Some 91.9% of participants chose to self-test following a demonstration and illustrated instructions.
	Choko et al. (63)	Uganda	Men	Single-arm pilot-trial of secondary distribution of HIVST kits	116 men	Seeds (peer distributors) distributed HIVST kits to men. Eighty-two percent of men accepted HIVST kits. Ninety-seven percent of recruited men and 100% of seeds reported being willing to recommend HIVST to their friends and family.
	Choko et al. (64)	Malawi	Pregnant women and male partners	Adaptive multi-arm, multi-stage cluster randomized trial	676 men; 2,349 women	Secondary distribution of HIVST kits provided by women to their male partners increased the proportion of men who tested and linkage to care and prevention services if accompanied by financial incentives and reminder calls.
	Gichangi et al. (65)	Kenya	Pregnant women and male partners	Randomized controlled trial	362 men; 387 women	Three-arm randomized control study of participants randomized to receive either standard-of-care plus standard information card, an information card referencing male HIV testing, or two oral HIVST kits, and HIV testing information. In the intervention group (arm 3), 82% of men reported HIV testing as a couple, compared with 28% in arm one and 37%in arm two.
	Hensen et al. (66)	Zambia	Men and women	Randomized controlled trial	3,677 men; 5,428 women	PopART intervention used door-to-door delivery of HTS and included HIVST. Uptake of secondary distribution of HIVST was 9.1%, of which, 55.8% of kits were reported to have been used.
	Kalibala et al. (67)	Kenya	HCWs	Semi-structured pretested questionnaire and in-depth interview	842 HCWs	Thirty-four of surveyed HCWs used the kit on themselves; seventy-three percent provided a kit to their partner.
	Kelvin et al. (68)	Kenya	Truck drivers	Randomized controlled trial	305 male truck drivers	Participants were recruited from two roadside wellness clinics in Kenya. Participants were randomized on a 1:1 basis to either the SOC arm (provider-administered FS test) or the Choice arm (choice of SOC test or self-administered oral rapid test). The Choice arm had significantly greater odds of testing uptake. Of those in the Choice arm who tested, 26.9% selected the SOC test, 64.6% chose supervised self-testing in the clinic, and 8.5% took a test kit for home use. Participants varied in the HIV test they selected when given choices.
	Kelvin et al. (69)	Kenya	Truck drivers	Randomized controlled trial	2,262 male truck drivers	Texting about the availability of HIVST kits increased testing rates from 1.3 to 3.5%.
	Kisa et al. (70)	Uganda	Pregnant women and male partners	Cross-sectional study nested within a cluster randomized HIVST trial	51 women; 44 men	Most participants (94.7%) underwent repeat HIVST with a returned 2.1% positivity rate.
	Kumwenda et al. (71)	Malawi	Men and women	In-depth interviews nested in a cluster randomized trial	33 participants	Community counselors provided HIVST to community members through a community-based model prior to the interviews. More men than women declined joint HIVST due to fear of their infidelity being exposed.
	Lippman et al. (72)	South Africa	MSM	Three-phase trial	133 MSM	Men were recruited over three phases (different locations) of which they were given HIVST kits. Errors were committed by persons in both the OF and FS group; however, participants successfully performed the OF test while FS was less consistent. FS was a more preferred option than OF.
	Lippman et al. (73)	South Africa	MSM	Longitudinal study	127 MSM	Men were given up to nine test kits, either OF or FS, to use themselves or to provide to their social networks. Almost all MSM (91%) self-tested. A majority of men (80%) preferred HIVST to testing at a clinic.
	Marwa et al. (74)	Kenya	Pregnant women and male partners	Randomized controlled trial	1,107 couples	Three-arm RCT of participants randomized to either arm one (SOC), arm two (letter of invitation for partner to test, and arm three (letter and instructions on how to use HIVST and two HIVSTs with counseling). Men in arm three were twelve times more likely to test when compared to arm one. improved male invitation letter increased the likelihood of male partner testing by twelve times.
	Masters et al. (10)	Kenya	Men and women	Randomized controlled trial	600 women	Participants were randomized in a 1:1 ratio using balanced block randomization to an HIVST group or a comparison group. Participants in the HIVST group received two oral-fluid-based rapid HIV tests alongside written instructions and a brief demonstration of how to use the test. Male partner HIV testing was higher (90.8 vs. 51.7%) among participants in the HIVST group. Couples testing was also more likely in this group (75.4 vs. 45.8%).
	Moore et al. (75)	South Africa	Men and women	Cohort study	33 men; 606 women	The sending of short message service (SMS) to participants aided participants in reporting HIVST results.
	Mugo et al. (76)	Kenya	Pharmacy clients	Exploratory feasibility study	225 men; 238 women	Staff at five pharmacies recruited clients and offered participants HIVST kits for $1 USD. Participants were contacted for post-test data collection and counseling. Almost all testers stated they would like to use HIVST again in the future, and that they were likely (19%) or very likely (80%) to recommend self-testing to a friend, partner or family member.
	Pintye et al. (77)	Kenya	Women and their male partners	Implementation project	3,620 women	Some 93% of women offered an HIVST to their male partner. Of those women, 95% of male partners used a self-test.
	Schaffer et al. (78)	Uganda	Men	Discrete choice experiment	203 men	When presented as a choice, distribution of HIVST kits at local pharmacies reported the lowest predicted uptake and was higher among men who perceive a higher relative risk of having HIV.
	Strauss et al. (79)	Kenya	Truck drivers	Discrete choice experiment	305 male truck drivers	Participants were presented with hypothetical options of making trade-offs between different characteristics of HIV testing services delivery models by making hypothetical choices in a series of paired HIV testing scenarios to identify which HIV testing characteristics influenced the selection of preferred options. Drivers who had previous testing experience preferred oral testing and counseling via telephone while drivers with no testing experience preferred clinic-based testing.
	Strauss et al. (80)	Kenya	Truck drivers	Randomized control trial	150 male truck drivers	Cost drove the preference of between self-testing and provider administered testing. Self-testers preferred oral-testing vs. finger-prick testing.
	Thirumurthy et al. (81)	Kenya	Women	Cohort study	280 participants	Study staff instructed one arm of women on how to use OF based rapid HIV tests and provided them multiple test kits. The other arm was given three test kits each and FSW IPs were given five test kits each. Ninety-one percent of women in antenatal care and 86% in post-partum care distributed HIVST kits to their primary sexual partners. Seventy-five percent of female sex workers distributed HIVST kits to their clients.

### HIVST Knowledge

Regarding knowledge of HIVST, in Uganda, researchers found that most participants had never heard of HIVST (23). In Tanzania, A lack of knowledge was found for HIVST with only 22% of participants having heard of HIVST (27). In another study, most healthcare users (HCU) (69.9%), consisting of both men and women reported having heard of HIVST in South Africa (30). Additionally, in South Africa, only 3.9% of men had ever heard of oral HIVST prior to the study (47). Lastly, less than half of students (46.2%) knew what HIVST was prior to the administration of a survey in South Africa (50).

### Acceptability and HIVST Benefits

In South Africa, preferences of testing were associated with patient autonomy, violation of human rights, confidentiality and privacy, fear of discrimination and stigma, and an aversion to mandatory face-to-face counseling with participants favoring HIVST for these reasons (57). One cross-sectional study from Malawi, Kenya, and South Africa reported that participants were in support of the idea of an accurate, easy-to-use, rapid HIVST and believed that this could increase testing across all populations (58). Furthermore, in Uganda, both men and women believed HIVST was a strategy that could address men's lack of time to go to the health facilities to test for HIV (49). In South Africa, in support of the testing method, HIVST was deemed acceptable (55). In Kenya the acceptability rate for HIVST was 94% (41). Both oral fluid (OF) and finger-stick (FS) methods had high acceptability of which ease of access and the availability of the test were cited as being of importance (38) and FS was preferred to OF tests, both in South Africa (72). In Kenya, self-testers preferred OF vs. FS (80). However, South African participants believed that the acceptability of home OF uptake would not necessarily lead to higher uptake of the test (46). Furthermore, participants also believed that men would show the most interest in using home OF compared to their female counterparts (46). In Zimbabwe, door-to-door delivery of kits was highly preferred compared to kit distribution to batch deliveries (54). After being informed about HIVST, 91% of participants reported being comfortable with using a self-test; 87% believed that HIVST would increase their likelihood of testing in Zambia (59). Still, naïve testers were confident in performing unsupervised HIVST but reported desiring more counseling support during the testing process in South Africa (32). South African men deemed HIVST acceptable because of its convenience and efficiency (33). In South Africa, participants deemed HIVST acceptable due to its privacy, ease of use, and trustworthiness (53). HIVST acceptability was high among the students (87.1%) (50). In South Africa, researchers found that participants had a high average usability index of 93.8% for HIVST (44). In Tanzania, participants reported positive attitudes toward HIVST, supportive perceived norms, and self-efficacy (51). In Malawi and Zambia, most women and male partners thought home-based testing and secondary distribution of HIV self-test kits were acceptable (36). In Uganda, women believed that HIVST could help to improve couples' HIV testing (48). In Kenya, truck drivers, who had previous testing experience, preferred oral testing and counseling via telephone while drivers with no testing experience preferred clinic-based testing (79). In Malawi and Zambia, of three male partner HIV testing strategies (HIV partner notification, partner HIV self-testing and partner home-based HIV testing) most participants (both men and women) accepted all three partner testing modalities; however, male partners were split in their preferences for the three partner testing modalities (36). Key stakeholders perceived HIVST as an effective initiative that may be used to increase uptake of testing services for underserved populations in Rwanda (29).

In South Africa, some 96.6% of participants found HIVST easy to use (44) and Individual motivations for HIVST included perceived benefits of access to treatment. HIVST was regarded as convenient, confidential, reassuring and an enabling new way to test with one's partner (39). In Senegal, most participants (74.5%) were comfortable using HIVST, 86.1% found the instructions easy to follow, and 94.4% believed their family or friends would use it (43). Participants in Uganda believed there were benefits associated with HIVST, such as privacy, convenience (55), and being able to test before sex (23). In Rwanda, most HCU (81.2%) perceived HIVST as a strategy that could lead to more people knowing their HIV status (30). In Malawi, individual motivations for HIVST included perceived benefits of access to treatment; HIVST was regarded as confidential, reassuring and as a novel way to test with one's partner (40). South African stakeholders, consisting of two government officials, four non-governmental organization stakeholders, two donors, three academic researchers, and one international stakeholder, expressed high enthusiasm regarding HIVST, its scale-up, and the development of HIVST policies and programming (45). Secondary distribution of HIVST kits was thought to be convenient, ensured confidentiality, allowed men to avoid the clinic, and allows for couples testing privately in Malawi and Zambia (36). Home-based testing was thought to be convenient and would provide savings in time and transport money, and helpful to have health workers present to provide counseling (36). In South Africa, 22% of participants preferred HIVST; however, 66% of participants (mostly men) preferred client-initiated testing (56). In Malawi, male partners reported a preference for HIVST due to its perceived privacy and reduction of associated stigma (26). In Kenya, almost all testers stated they would like to use HIVST again in the future, and that they were likely (19%) or very likely (80%) to recommend self-testing to a friend, partner or family member (76). Also, 97% of recruited men and 100% of seeds (peer distributors) reported being willing to recommend HIVST to their friends and family in Malawi (63).

In South Africa, a smartphone app used in tandem with an oral HIVST was able to help people through the self-testing process by providing counseling and care and simplifying the process of self-testing (37). The app was able to multiple common HIV testing barriers, such as lack of confidentiality, wait times, and testing locations (37). The app also enabled testing services outside a clinic context or within a clinic; however, an additional layer of privacy was added by using the app (37). Participants were able to use the app based HIVST strategy unsupervised at home, unsupervised alone at the Kiosk around the clinic, or supervised under direct supervision of staff at the clinic (37).

### Willingness to Use HIVST

In Tanzania, 65% of participants were willing to use HIVST in the future (27) and HIVST willingness was high among both male ever-testers and never-testers with 72% of ever-testers vs. 67% of never-testers reported being willing to self-test (28). Most women were willing to take the kits to their male partners and male partners reported being willing to use HIVST kits provided to them by their female partners in Uganda (48). Participants reported being willing to use HIVST if it included telephone counseling and if it were available in their communities in South Africa (56). Three-quarters of students were willing to purchase an HIVST kit and many reported being willing to self-test with their partners in South Africa (50).

### Uptake

Broadly, several community-based HIVST interventions throughout sub-Saharan Africa reported that the most significant impact has been with adult men, with an average of 1,500 HIV infections averted (24). HIVST was found to be advantageous when provided in combination with existing services, which resulted in several distribution models having high male participation in Malawi, Zambia, and Zimbabwe (34). Uptake of oral HIVST was reported as 25.4% in South Africa (47). One study conducted in Malawi reported that 76.5% of residents self-tested during a 12-month period (25). In South Africa, almost all men who have sex with men (MSM) (91%) self-tested and most men (80%) preferred HIVST to testing at a clinic (73). Furthermore, an overall estimated uptake of HIVST >80% was reported with uptake being greater among women than men (25). Also, 91.9% of participants chose to self-test following a demonstration and illustrated instructions (62). Both accessibility and availability to HIVST were influential to uptake as described in a study from Ethiopia (38).

Finally, both unsupervised and supervised HIVST were preferred across sub-Saharan populations for varying reasons. In Johannesburg, South Africa, it was reported that 68.7% of participants selected unsupervised HIVST, while 25% opted for supervised HIVST and 6.3% chose semi-supervised (42). In Mozambique, when asked to choose a test to be administered, over 80% of participants selected to perform directly assisted HIVST compared to standard FS testing and of those who selected HIVST, 20% opted to perform HIVST at home (35). Still, more than three-fourths of participants (76%) opted to perform HIVST at the health center due to the presence of a counselor (35).

### HIVST Barriers

In Uganda, perceived barriers for HIVST included the absence of professional support, poor disposal of kits, and delayed linkage to care (23). Participants in Malawi sometimes required persuasion even though they believe HIVST is more flexible than traditional testing (40). Perceived barriers included a lack of counseling and difficulty in ensuring linkages to care among those with positive results in South Africa (45). Participants had concerns (potential suicidality) regarding the lack of HIV counseling associated with HIVST in South Africa (55). Participants from Central Uganda reported skepticism regarding HIVST and whether the kits could actually test for HIV, but this was not a deterrent to its utilization (49). Also, in a cross-sectional study from Malawi, Kenya, and South Africa, it was reported that participants found instructions for HIVST to be confusing and/or difficult to follow (52). Less than 25% of participants completed the test successfully without errors (52). Results interpretation was difficult for participants (52). In South Africa, errors were committed by participants in both the oral fluid (OF) and finger stick (FS) group; however, most participants successfully performed the OF test while FS was less consistent. Lastly, in 10% of participants needed help or made errors while using HIVST kits in Malawi (62).

## Intervention Strategies

Twenty-four articles reported various intervention strategies regarding HIVST. Strategies included choices of testing strategies, cost/financial incentives, distribution strategies, and miscellaneous strategies.

### Choices/Options of Testing

In Kenya, participants were presented with options of making trade-offs between different characteristics of HIV testing service delivery models by making hypothetical choices in a series of paired HIV testing scenarios to identify which HIV testing characteristics influenced the selection of preferred options (79). In Uganda, when presented as a choice, distribution of HIVST kits at local pharmacies reported the lowest predicted uptake and was higher among men who perceive a higher relative risk of having HIV (78).

Participants were recruited from two roadside wellness clinics in Kenya and were randomized on a 1:1 basis to either the standard of care (SOC) arm (provider-administered FS test) or the Choice arm (choice of SOC test or self-administered oral rapid test) (68). The Choice arm had significantly greater odds of testing uptake. Of those in the Choice arm who tested, 26.9% selected the SOC test, 64.6% chose supervised self-testing in the clinic, and 8.5% took a test kit for home use. Therefore, participants varied in the HIV test they selected when given choices (68). In South Africa, MSM were recruited over three phases (different locations) when they were given HIVST kits (72). Still, texting about the availability of HIVST kits increased testing rates from 1.3 to 3.5% in Kenya (69). Furthermore, the sending of short message service (SMS) to participants aided participants in reporting HIVST results in South Africa (75).

### Cost/Financial Incentives

Free HIVST kits were preferred compared to kits available to be purchased by male and female regular testers to overcome the financial burden associated with obtaining HTS, a prominent deterrent for linkage to care in several developing sub-Saharan nations (15). Cost (free vs. paid) drove the preference between self-testing and provider administered testing in Kenya (80). In Zimbabwe, participants were provided vouchers to be redeemed for HIVST within 1 month at prices between $0 and $3 at multiple sites (61). A high sensitivity to price for HIVST was realized among men, rural residents, and persons who had never tested for HIV, while reduced-priced or free tests increased HIVST demand (61). Also, in Zimbabwe, the strongest preference for kits was price—every $1 increase in price increased disutility (54). Staff at five pharmacies recruited clients and offered participants HIVST kits for $1 USD in Kenya (76). In one paper, the main theme in the behavioral study was affordability; participants were willing to pay up to 111 Kenyan shillings (Ksh) (around $1.25 USD) for an HIVST kit (41).

### HIVST Distribution Strategies

In Uganda, seeds (peer distributors) distributed HIVST kits to men; 82% of men accepted HIVST kits from their peers (63). Community counselors provided HIVST to community members through a community-based model prior to the interviews in Malawi (71). More men than women declined joint HIVST due to fear of their infidelity being exposed (71). In South Africa, MSM were given up to nine test kits, either OF or FS, to use themselves or to provide to their social network (73). In Kenya, 93% of women offered an HIVST to their male partner and of those women, 95% of male partners used a self-test (77). Hospitals were randomly selected from each region of Kenya (67). Thirty-four of surveyed HCWs used the kit on themselves; 73% provided a kit to their partner in Kenya (67). Also in Kenya, staff instructed two arms of women from antenatal and postpartum clinics on how to use OF based rapid HIV tests and provided them multiple test kits; the women were given three test kits each and FSWs were given five test kits each (81). Ninety-one percent of women in antenatal care and 86% in post-partum care distributed HIVST kits to their sexual partners. Seventy-five percent of female sex workers distributed HIVST kits to their clients (81). Secondary distribution of HIVST kits provided by women to their male partners increased the proportion of men who tested, linkage to care, and prevention services if accompanied by financial incentives and reminder calls (64). In Zambia, door-to-door delivery of HIV Testing Services (HTS) was offered to participants and included HIVST (66).

In a three-arm RCT, participants were randomized to either arm one [standard of care (SOC)], arm two (letter of invitation for partner to test), and arm three (letter and instructions on how to use HIVST and two HIVSTs with counseling) in Kenya (74). Men in arm three were twelve times more likely to test when compared to arm one; improved male invitation letter increased the likelihood of male partner testing by twelve times (74). Also, in Kenya, a three-arm RCT of participants randomized to receive either SOC plus standard information card, an information card referencing male HIV testing, or two oral HIVST kits, and HIV testing information (65). In the intervention group (arm 3), 82% of men reported HIV testing as a couple, compared with 28% in arm one and 37% in arm two (65). Uptake of secondary distribution of HIVST was 9.1%, of which, 55.8% of kits were reported to have been used (66).

### Miscellaneous Strategies

In Malawi, participants received pre-test counseling, instructions on how to perform HIVST, and were asked to demonstrate their understanding of how to use the kit (25). Participants were also offered self-testing plus confirmatory HTC (parallel testing with two rapid finger-prick blood tests), standard HTC alone, or no testing (62). In Uganda, participants were offered HIVST or standard of care in a cluster randomized HIVST trial; most participants (94.7%) underwent repeat HIVST with a returned 2.1% positivity rate after having used the kits (70) In Uganda, participants were randomized to either an unsupervised HIVST group or a provider supervised HIVST group; unsupervised HIVST able to identify 90% of HIV-infected persons (60).

## Discussion

We completed a systematic review to assess published articles regarding HIVST uptake and intervention strategies among men in sub-Saharan Africa. Though more attention and research has been paid to HIVST in recent years, men in sub-Saharan Africa are still not testing at rates consistent with their female counterparts. The intervention strategies found in this review aimed to increase HIV testing for men in some capacity. Novel approaches, such as the targeting of truck drivers who are at high risk of HIV infection at truck stops in Kenya (80) offer the chance of accessing such a hard-to-reach niche group. Still, other strategies have been used successfully as well. In a study ineligible for inclusion in this review, community health counselors have been used to target hard-to-reach populations as well as reported in a recent a study (82). To sustain awareness of the availability of HIVST, counselors consistently sensitized their communities through the distribution of flyers and regular interaction with potential clients (82). Over a 12-month period, counselors achieved over 80% adult uptake of HIVST within their respective cluster (82). Neuman et al. (83) developed a protocol for HIVST to be provided by trained lay distributors selected by the community. Trials evaluated the effectiveness of distribution of HIVST kits by community-based distribution agents on uptake of HIV testing (83). Strategies, such as these are necessary for increasing the uptake of HIVST. There is no one intervention strategy that will work universally for meeting the needs of all men in the sub-Saharan Africa region. It is important that multiple strategies be employed in several locations in order to better locate men and provide what they need to be tested for HIV.

Overall, HIVST was found to be acceptable and when surveyed, most participants reported being willing to either use HIVST kits themselves and/or recommend it to family and friends. Research has provided further evidence of the acceptability of HIVST. A study, not included in this review, found that 96% of participants reported that they would use a self-test if it were available to them and 95.5% would recommend a self-test to their sexual partners (84). Secondary distribution of HIVST kits to men by peers or their partners were highlighted in this review and are advantageous ways to reach some men. These avenues of HIVST kit distribution should continue to be utilized. HIVST kit distribution via female sex workers should also be prioritized. Testing kits should also be provided for free to all users in order to address the barriers of cost that play a role in men not utilizing HIVST.

One of the major findings of this review is that HIVST is deemed to be more convenient than traditional testing. Yet, there were also findings of some men being skeptical of HIVST and were not convinced of the accuracy of the kits (49). There were also issues identified with persons being unable to complete their HIVST without errors (52). These findings suggest that there is a great need for more health information pertaining to HIVST and its benefits as well as its accuracy to be provided in the region. There is also a need for the instructions which accompany each HIVST kit to be reviewed (85) and tested among diverse populations in the region. Pre-test counseling, as mentioned in Choko et al. (25) article, is also worthy of further exploration in order to minimize user error. Overall, HIV counseling in general is still needed and should be provided to men who opt to use HIVST. In the context of self-testing, HIV counseling is necessary for addressing concerns around testing, stressing the importance of confirmatory testing, and achieving linkage to care for those testing positive. HIV counseling may also be completed via SMS or a mobile app in order for testers to report their HIVST results and schedule a convenient date and time for in-depth counseling, receive a confirmatory HIV test, and be linked to care (86). Using home visits or phone, or through a mobile app, HIVST can be better promoted as convenient and efficient. Lastly, as pre-exposure prophylaxis (PrEP) becomes more available in the region, men who engage in risky-sexual behaviors should be provided PrEP to add to the current toolbox of HIV prevention methods.

Further research should investigate the use of HIVST and linkage to care in tandem for men in sub-Saharan Africa. Linking men to care is paramount to reducing HIV incidence in the region. Past research reported some 85% of respondents being willing to link to care following a positive test (86). Respondents also preferred home visits or phone calls to SMS for linking to care (86). Another study reported that linkage to care for participants was estimated to be 56.3% (524/930) (25). Furthermore, it was reported that over 97% of men reported using the HIVST kit at 3-month follow up (87). All participants who tested positive (5.6%) sought a confirmatory test and began HIV treatment (87). Linkage to care was confirmed by participants via 8.7% receiving counseling, 16% initiated ART, and 5.3% CD4-tested (42). Future research should also investigate the use of multiple venues as a means for reaching men in sub-Saharan Africa. Past research has reported uptake of HIVST being high for both the home-based (64.9%) and facility-based groups (52.7%) (88). Significantly, more adults reported positive HIVST results in the home group (6.0%) vs. the facility group, (3.3%) (88).

This systematic review is subject to limitations that should be considered. First, only full-text peer-reviewed articles that were written in English were included. Also, while this review aimed to review published articles pertaining to HIVST uptake and intervention strategies among men in sub-Saharan Africa, articles meeting our criteria included both men and women. Furthermore, country specific names and truncated regional names were not used during the database search; this search strategy may have omitted relevant articles. Study evaluations, and cost analyses were also not included. Articles which fit into the categories may have provided salient strategies for increasing HIVST uptake. One article was omitted due to it not meeting the required age restriction of participants being 16 years or older; the population of interest included participants as young as. Finally, only articles which were published between 2010 and 2020 were included in this review. Still, this review has multiple strengths which is necessary to highlight. First, the various quality assessment tools used for article evaluation ensures that included articles' design, analysis, and reporting has been properly considered and carried out and indicates the quality of included studies. Also, the rigor of the methodology used in this review presents an accurate and comprehensive account of articles pertaining to HIVST uptake and intervention strategies among men in sub-Saharan Africa.

## Conclusion

HIVST is highly acceptable to men. More efforts are needed to develop policies to implement HIVST programs targeting men in Sub-Saharan Africa, including a focus on linkage to care in sub-Saharan Africa. Future interventions should directly target men independently in tandem with using peers and their romantic partners to promote self-testing among men in sub-Saharan Africa. HIVST kit distribution strategies should be combined with services that can offer confirmatory tests and counseling for men as well as linkage to care. The continuation of implementing health education, promotion, and the offering of HIVST at multiple venues and target areas where men in each country are known to congregate is necessary. Country-specific HIVST intervention strategies and methods are also necessary to achieve the greatest reach. Lastly, PrEP strategies for men in tandem with HIVST should developed and implemented in countries where PrEP is available.

## Data Availability Statement

The original contributions presented in the study are included in the article/supplementary material, further inquiries can be directed to the corresponding author/s.

## Author Contributions

AH and NT completed the data extraction quality assessment, and manual searches for articles. AC, MH, PJ, JK, and DC reviewed, edited, and provided the insight for the development of the manuscript. All authors contributed to the article and approved the submitted version.

## Conflict of Interest

The authors declare that the research was conducted in the absence of any commercial or financial relationships that could be construed as a potential conflict of interest.

## References

[1] World Health Organization. Global Health Observatory Data. (2019). Available online at: https://www.who.int/gho/hiv/en/ (accessed May 5, 2020).

[2] UNAIDS. Ending AIDS: Progress Towards the 90–90–90 Targets. (2017). Available online at: https://www.unaids.org/en/resources/documents/2017/20170720_Global_AIDS_update_2017 (accessed May 5, 2020).

[3] Musheke M Ntalasha H Gari S McKenzie O Bond V Martin-Hilber A. A systematic review of qualitative findings on factors enabling and deterring uptake of HIV testing in Sub-Saharan Africa. BMC Public Health. (2013) 13:220. 10.1186/1471-2458-13-220 23497196 PMC3610106

[4] Haroz D von Zinkernagel D Kiragu K. Development and impact of the global plan. J Acquir Immune Defic Syndr. (2017) 75:S2–6. 10.1097/QAI.0000000000001318 28398991 PMC5400405

[5] UNAIDS. Blind Spot: Reaching Out to Men and Boys. (2017). Available online at: https://www.unaids.org/sites/default/files/media_asset/blind_spot_en.pdf (accessed May 5, 2020).

[6] Tanzania Commission for AIDS Zanzibar AIDS Commission National Bureau of Statistics Office of Chief Government Statistician ICF International. Tanzania HIV/AIDS and Malaria Indicator Survey 2011–12. (2013). Available online at: https://dhsprogram.com/pubs/pdf/AIS11/AIS11.pdf (accessed April 7, 2020).

[7] International Center for AIDS Care and Treatment Programs. A Population-Based HIV Impact Assessment (PHIA) 2016–2017: Tanzania HIV Impact Survey (THIS). (2018). Available online at: https://phia.icap.columbia.edu/wp-content/uploads/2016/09/THIS_Final.pdf (accessed May 10, 2020).

[8] UNITAID World Health Organization. Market and Technology Landscape: HIV Rapid Diagnostic Tests for Self-Testing: Fourth Edition. (2018). Available online at: https://www.who.int/hiv/pub/self-testing/hiv-self-testing-2018-edition4/en/ (accessed May 5, 2020).

[9] World Health Organization Unitaid HIV Self-testing Africa Initiative. Knowing Your Status–Then and Now. (2018). Available online at: https://www.who.int/hiv/pub/vct/who-unitaid-know-your-hiv-status/en/ (accessed May 5, 2020).

[10] Masters SH Agot K Obonyo B Napierala M Maman S Thirumurthy H. Promoting partner testing and couples testing through secondary distribution of HIV self-tests: a randomized clinical trial. PLoS Med. (2016) 13:e1002166. 10.1371/journal.pmed.1002166 27824882 PMC5100966

[11] Mulubwa C Hensen B Phiri MW Shanaube K Schaap AJ Floyd S. Community based distribution of oral HIV self-testing kits in Zambia: a cluster-randomised trial nested in four HPTN 071 (PopART) intervention communities. Lancet HIV. (2019) 6:e81–92. 10.1016/S2352-3018(18)30258-3 30584047 PMC6361868

[12] Estem KS Catania J Klausner JD. HIV self-testing: a review of current implementation and fidelity. Curr HIV/AIDS Rep. (2016) 13:107–15. 10.1007/s11904-016-0307-y 26879653

[13] Johnson CC Kennedy C Fonner V Siegfried N Figueroa C Dalal S. Examining the effects of HIV self-testing compared to standard HIV testing services: a systematic review and meta-analysis. J Int AIDS Soc. (2017) 20:21594. 10.7448/IAS.20.1.21594 28530049 PMC5515051

[14] Stevens DR Vrana CJ Dlin RE Korte JE. A global review of HIV self-testing: themes and implications. AIDS Behav. (2018) 22:497–512. 10.1007/s10461-017-1707-8 28155039 PMC5910655

[15] Njau B Covin C Lisasi E Damian D Mushi D Boulle A. A systematic review of qualitative evidence on factors enabling and deterring uptake of HIV self-testing in Africa. BMC Public Health. (2019) 19:1289. 10.1186/s12889-019-7685-1 31615461 PMC6794839

[16] Hlongwa M Mashamba-Thompson T Makhunga S Hlongwana K. Mapping evidence of intervention strategies to improving men's uptake to HIV testing services in sub-Saharan Africa: a systematic scoping review. BMC Infect Dis. (2019) 19:496. 10.1186/s12879-019-4124-y 31170921 PMC6554953

[17] Hlongwa M Mashamba-Thompson T Makhunga S Muraraneza C Hlongwana K. Men's perspectives on HIV self-testing in sub-Saharan Africa: a systematic review and meta-synthesis. BMC Public Health. (2020) 20:66. 10.1186/s12889-020-8184-0 31941479 PMC6964071

[18] Moher D Liberati A Tetzlaff J Altman DG. Preferred reporting items for systematic reviews and meta-analyses: the PRISMA statement. PLoS Med. (2009) 6:e1000097. 10.1371/journal.pmed.1000097 19621072 PMC2707599

[19] Critical Appraisal Skills Programme UK. CASP Checklists. (2020). Available online at: https://casp-uk.net/casp-tools-checklists/ (accessed May 25, 2020).

[20] National Institutes of Health. Quality Assessment Tool for Observational Cohort and Cross-Sectional Studies. (2014). Available online at: https://www.nhlbi.nih.gov/health-topics/study-quality-assessment-tools (accessed May 5, 2020).

[21] Sterne JAC Savović J Page MJ Elbers RG Blencowe NS Boutron I. RoB 2: a revised tool for assessing risk of bias in randomised trials. BMJ. (2019) 366:l4898. 10.1136/bmj.l4898 31462531

[22] Popay J Roberts H Sowden A Petticrew M Arai L Rodgers M. Guidance on the Conduct of Narrative Synthesis in Systematic Reviews. (2006). Available online at: https://www.researchgate.net/profile/Mark_Rodgers4/publication/233866356_Guidance_on_the_conduct_of_narrative_synthesis_in_systematic_reviews_A_product_from_the_ESRC_Methods_Programme/links/02e7e5231e8f3a6183000000/Guidance-on-the-conduct-of-narrative-synthesis-in-systematic-reviews-A-product-from-the-ESRC-Methods-Programme.pdf (accessed May 5, 2020).

[23] Burke VM Nakyanjo N Ddaaki W Payne C Hutchinson N Wawer MJ. HIV self-testing values and preferences among sex workers, fishermen, and mainland community members in Rakai, Uganda: a qualitative study. PLoS ONE. (2017) 12:e0183280. 10.1371/journal.pone.0183280 28813527 PMC5558930

[24] Cambiano V Johnson CC Hatzold K Terris-Prestholt F Maheswaren H Thirumurthy H. The impact and cost-effectiveness of community-based HIV self-testing in sub-Saharan Africa: a health economic and modelling analysis. J Int AIDS Soc. (2019) 22:e25243. 10.1002/jia2.25243 30907498 PMC6432108

[25] Choko AT MacPherson P Webb EL Willey BA Feasy H Sambakunsi R. Uptake, accuracy, safety, and linkage into care over two years of promoting annual self-testing for HIV in Blantyre, Malawi: a community-based prospective study. PLoS Med. (2015) 12:e1001873. 10.1371/journal.pmed.1001873 26348035 PMC4562710

[26] Choko AT Kumwenda MK Johnson CC Sakala DW Chikalipo MC Fielding K. Acceptability of woman-delivered HIV self-testing to the male partner, and additional interventions: a qualitative study of antenatal care participants in Malawi. J Int AIDS Soc. (2017) 20:21610. 10.7448/IAS.20.1.21610 28691442 PMC5515040

[27] Conserve DF Muessig KE Maboko LL Shirima S Kilonzo MN Maman S. Mate Yako Afya Yako: formative research to develop the Tanzania HIV self-testing education and promotion (Tanzania STEP) project for men. PLoS ONE. (2018) 13:e0202521. 10.1371/journal.pone.0202521 30148846 PMC6110473

[28] Conserve DF Bay C Kilonzo MN Makyao NE Kajula L Maman S. Sexual and social network correlates of willingness to self-test for HIV among ever-tested and never-tested men: implications for the Tanzania STEP project. AIDS Care. (2018) 31:169–76. 10.1080/09540121.2018.1537466 30362377 PMC7295037

[29] Dzinamarira T Kamanzi C Mashamba-Thompson TP. Key stakeholders' perspectives on implementation and scale up of HIV self-testing in Rwanda. Diagnostics (Basel). (2020) 10:194. 10.3390/diagnostics10040194 32244566 PMC7235833

[30] Gumede SD Sibiya MN. Health care users' knowledge, attitudes and perceptions of HIV self-testing at selected gateway clinics at eThekwini district, KwaZulu-Natal province, South Africa. J. Soc. Aspects HIV/AIDS. (2018) 15:103–9. 10.1080/17290376.2018.1517607 PMC6127809 30175655

[31] Harichund C Karim QA Kunene P Simelane S Moshabela M. Exploring factors that influence the integration of HIVST with HCT using a qualitative comparative cross-over design in KwaZulu-Natal, South Africa. Glob Public Health. (2019) 14:1275–87. 10.1080/17441692.2019.1587638 30829120

[32] Harichund C Karim QA Kunene P Simelane S Moshabela M. HIV self-testing as part of a differentiated HIV testing approach: exploring urban and rural adult experiences from KwaZulu-Natal, South Africa using a cross-over study design. BMC Public Health. (2019) 19:53. 10.1186/s12889-018-6366-9 30634943 PMC6329077

[33] Harichund C Moshabela M Kunene P Karim QA. Acceptability of HIV self-testing among men and women in KwaZulu-Natal, South Africa. AIDS Care. (2019) 31:186–92. 10.1080/09540121.2018.1503638 30058362

[34] Hatzold K Gudukeya S Mutseta MN Chilongosi R Nalubamba M Nkhoma C. HIV self-testing: breaking the barriers to uptake of testing among men and adolescents in sub-Saharan Africa, experiences from STAR demonstration projects in Malawi, Zambia and Zimbabwe. J Int AIDS Soc. (2019) 22:e25244. 10.1002/jia2.25244 30907505 PMC6432104

[35] Hector J Davies M Dekker-Boersema J Aly AA Abdalad CCA Langa EBR. Acceptability and performance of a directly assisted oral HIV self-testing intervention in adolescents in rural Mozambique. PLoS ONE. (2018) 13:e0195391. 10.1371/journal.pone.0195391 29621308 PMC5886533

[36] Hershow RB Zimba C Mweemba O Chibwe KF Twambilile P Dunda W. Perspectives on HIV partner notification, partner HIV self-testing and partner home-based HIV testing by pregnant and postpartum women in antenatal settings: a qualitative analysis in Malawi and Zambia. J Int AIDS Soc. (2019) 22:e25293. 10.1002/jia2.25293 31321884 PMC6639664

[37] Janssen R Engel N Esmail A Oelofse S Krumeich A Dheda K. Alone but supported: a qualitative study of an HIV self-testing app in an observational cohort study in South Africa. AIDS Behav. (2019) 24:467–74. 10.1007/s10461-019-02516-6 31049809 PMC6989648

[38] Kebede B Abate T Desalew M. HIV self-testing practices among Health Care Workers: feasibility and options for accelerating HIV testing services in Ethiopia. Pan Afr Med J. (2013) 15:50. 10.11604/pamj.2013.15.50.2328 24106578 PMC3786151

[39] Knight L Makusha T Lim J Peck R Taegtmeyer M van Rooyen H. “I think it is right”: a qualitative exploration of the acceptability and desired future use of oral swab and finger-prick HIV self-tests by lay users in KwaZulu-Natal, South Africa. BMC Res Notes. (2017) 10:486. 10.1186/s13104-017-2810-7 28923121 PMC5604290

[40] Kumwenda MK Munthali A Phiri M Mwale D Gutteberg T MacPherson . Factors shaping initial decision-making to self-test amongst cohabiting couples in urban Blantyre, Malawi. AIDS Behav. (2014) 18:S396–404. 10.1007/s10461-014-0817-9 24929834 PMC4102820

[41] Kurth AE Cleland CM Chhun N Sidle JE Were E Nannyu V. Accuracy and acceptability of oral fluid HIV self-testing in a general adult population in Kenya. AIDS Behav. (2016) 20:870–9. 10.1007/s10461-015-1213-9 26438487 PMC4799243

[42] Lebina L Seatlholo N Taruberekera N Radebe M Kinghorn A Meyer T. Feasibility of community-based HIV self-screening in South Africa: a demonstration project. BMC Public Health. (2019) 19:898. 10.1186/s12889-019-7122-5 31286953 PMC6615295

[43] Lyons CE Coly K Bowring AL Liestman B Diouf D Wong VJ. Use and acceptability of HIV self-testing among first-time testers at risk for HIV in Senegal. AIDS Behav. (2019) 23:130–41. 10.1007/s10461-019-02552-2 31197701 PMC6773816

[44] Majam M Mazzola L Rhagnath N Lalla-Edward ST Mahomed R Venter WDF. Usability assessment of seven HIV self-test devices conducted with lay-users in Johannesburg, South Africa. PLoS ONE. (2020) 15:e0227198. 10.1371/journal.pone.0227198 31935228 PMC6959591

[45] Makusha T Knight L Taegtmeyer M Tulloch O Davids A Lim J. HIV self-testing could “revolutionize testing in South Africa, but it has got to be done properly”: perceptions of key stakeholders. PLoS ONE. (2015) 10:e0122783. 10.1371/journal.pone.0122783 25826655 PMC4380342

[46] Martínez Pérez G Cox V Ellman T Moore A Patten G Shroufi A. ‘I know that I do have HIV but nobody saw me': oral HIV self-testing in an informal settlement in South Africa. PLoS ONE. (2016) 11:e0152653. 10.1371/journal.pone.0152653 27044006 PMC4820175

[47] Martínez-Pérez G Steele SJ Govender I Arellano G Mkwamba A Hadebe M. Supervised oral HIV self-testing is accurate in rural KwaZulu-Natal, South Africa. Trop Med Int Health. (2016) 21:759–67. 10.1111/tmi.12703 27098272

[48] Matovu J Buregyeya E Arinaitwe J Wanyenze RK. ‘…if you bring the kit home, you [can] get time and test together with your partner': Pregnant women and male partners' perceptions regarding female partner-delivered HIV self-testing in Uganda – a qualitative study. Int J Std AIDS. (2017) 28:1341–7. 10.1177/0956462417705800 28449628

[49] Matovu J Kisa R Buregyeya E Chemusto H Mugerwa S Musoke W. ‘If I had not taken it [HIVST kit] home, my husband would not have come to the facility to test for HIV': HIV self-testing perceptions, delivery strategies, and post-test experiences among pregnant women and their male partners in Central Uganda. Global Health Action. (2018) 11:1–11. 10.1080/16549716.2018.1503784 30092155 PMC6095038

[50] Mokgatle MM Madiba S. High acceptability of HIV Self-Testing among technical vocational education and training college students in Gauteng and North West Province: what are the implications for the scale pp in South Africa? PLoS ONE. (2017) 12:e0169765. 10.1371/journal.pone.0169765 28141858 PMC5283675

[51] Njau B Covin C Lisasi E Damian D Mushi D Boulle A. Feasibility of an HIV self-testing intervention: a formative qualitative study among individuals, community leaders, and HIV testing experts in northern Tanzania. BMC Public Health. (2020) 20:490. 10.1186/s12889-020-08651-3 32293370 PMC7161285

[52] Peck RB Lim JM van Rooyen H Mukoma W Chepuka L Bansil P. What should the ideal HIV self-test look like? A usability study of test prototypes in unsupervised HIV self-testing in Kenya, Malawi, and South Africa. AIDS Behav. (2014) 18:S422–32. 10.1007/s10461-014-0818-8 24947852

[53] Ritchwood TD Selin A Pettifor A Lippman SA Gilmore H Kimaru L. HIV self-testing: South African young adults' recommendations for ease of use, test kit contents, accessibility, and supportive resources. BMC Public Health. (2019) 19:123. 10.1186/s12889-019-6402-4 30696422 PMC6352366

[54] Sibanda EL d'Elbee M Maringwa G Ruhode N Tumushime M Madanhire C. Applying user preferences to optimize the contribution of HIV self-testing to reaching the “first 90” target of UNAIDS Fast-track strategy: results from discrete choice experiments in Zimbabwe. J Int AIDS Soc. (2019) 22:e25245. 10.1002/jia2.25245 30907515 PMC6432101

[55] Spyrelis A Abdulla S Frade S Meyer T Mhazo M Meyer T. Are women more likely to self-test? A short report from an acceptability study of the HIV self-testing kit in South Africa. AIDS Care. (2017) 29:339–43. 10.1080/09540121.2016.1234687 27654217

[56] van Dyk AC. Client-initiated, provider-initiated, or self-testing for HIV: what do South Africans prefer? JANAC. (2013) 24:e45–56. 10.1016/j.jana.2012.12.005 23582579

[57] van Dyk AC. Self-testing as strategy to increase the uptake of HIV testing in South Africa. AJAR. (2013) 12:41–8. 10.2989/16085906.2013.815413 25871310

[58] van Rooyen H Tulloch O Mukoma W Makusha T Chepuka L Knight LC. What are the constraints and opportunities for HIVST scale-up in Africa? Evidence from Kenya, Malawi and South Africa. J Int AIDS Soc. (2015) 18:19445. 10.7448/IAS.18.1.19445 25797344 PMC4369555

[59] Zanoli A Chipungu J Vinikoor MJ Bosomprah S Mafwenko M Holmes CB. HIV self-testing in Lusaka Province, Zambia: acceptability, comprehension of testing instructions, and individual preferences for self-test kit distribution in a population-based sample of adolescents and adults. AIDS Res Hum Retroviruses. (2018) 34:254–60. 10.1089/aid.2017.0156 28969432 PMC5863088

[60] Asiimwe S Oloya J Song X Whalen CC. Accuracy of un-supervised versus provider-supervised self-administered HIV testing in Uganda: A randomized implementation trial. AIDS Behav. (2014) 18:2477–84. 10.1007/s10461-014-0765-4 24691923 PMC4183743

[61] Chang W Matambanadzo P Takaruza A Hatzgold K Cowan FM Sibanda E. Effect of prices, distribution strategies, and marketing demand for HIV self-testing in Zimbabwe: a randomized clinical trial. JAMA Network Open. (2019) 2:e199818. 10.1001/jamanetworkopen.2019.9818 31461146 PMC6716290

[62] Choko AT Desmond N Webb EL Chavula K Napierala-Mavedzenge S. The uptake and accuracy of oral kits for HIV self-testing in high HIV prevalence setting: a cross-sectional feasibility study in Blantyre, Malawi. PLoS Med. (2011) 8:e1001102. 10.1371/journal.pmed.1001102 21990966 PMC3186813

[63] Choko AT Nanfuka M Birungi J Teasi G Kisembo P Helleringer S. A pilot trial of the peer-based distribution of HIV self-test kits among fishermen in Bulisa, Uganda. PLoS ONE. (2018) 13:e0208191. 10.1371/journal.pone.0208191 30496260 PMC6264512

[64] Choko AT Corbett EL Stallard N Maheswaran H Lepine A Johnson CC. HIV self-testing alone or with additional interventions, including financial incentives, and linkage to care or prevention among male partners of antenatal care clinic attendees in Malawi: an adaptive multi-arm, multi-stage cluster randomised trial. PLoS Med. (2019) 16:e1002719. 10.1371/journal.pmed.1002719 30601823 PMC6314606

[65] Gichangi A Wambua J Mutwiwa S Njogu R Bazant E Wamicwe J. Impact of HIV self-test distribution to male partners of ANC clients: results of a randomized controlled trial in Kenya. J Acquir Immune Defic Syndr. (2018) 79:467–73. 10.1097/QAI.0000000000001838 30148731 PMC6250253

[66] Hensen B Schaap AJ Mulubwa C Floyd S Shanaube K Phiri MM. Who accepts and who uses community-based secondary distribution HIV self-testing (HIVST) kits? Findings from the intervention arm of a cluster-randomized trial of HIVST distribution nested in four HPTN 071 (PopART) communities in Zambia. J Acquir Immune Defic Syndr. (2020) 84:355–64. 10.1097/QAI.0000000000002344 32195749 PMC7340225

[67] Kalibala S Tun W Cherutich P Nganga A Oweya E Oluouch P. Factors associated with acceptability of HIV self-testing among healthcare workers in Kenya. AIDS Behav. (2014) 18:S405–14. 10.1007/s10461-014-0830-z 24974123 PMC4933836

[68] Kelvin EA George G Mwai E Nyaga E Mantrell JE Romo ML. Offering self-administered oral HIV testing to truck drivers in Kenya to increase testing: a randomized controlled trial. AIDS Care. (2018) 30:47–55. 10.1080/09540121.2017.1360997 28826229 PMC5901679

[69] Kelvin EA George G Kinyanjui S Mwai E Romo ML Oruko F. Announcing the availability of oral HIV self- test kits via text message to increase HIV testing among hard-to-reach truckers in Kenya: a randomized controlled trial. BMC Public Health. (2019) 19:7. 10.1186/s12889-018-6345-1 30606161 PMC6318910

[70] Kisa R Matovu JKB Buregyeya E Musoke W Vrana-Diaz CJ Korte JE. Repeat HIV testing of individuals with discrepant HIV self-test results in Central Uganda. AIDS Res Ther. (2019) 16:26. 10.1186/s12981-019-0243-1 31514745 PMC6739989

[71] Kumwenda MK Corbett EL Chikovore J Phiri M Mwale D Choko AT. Discordance, disclosure, and normative gender roles: barriers to couple testing within a community-level HIV self-testing intervention in urban Blantyre, Malawi. AIDS Behav. (2018) 22:2491–9. 10.1007/s10461-018-2038-0 29411227 PMC6097721

[72] Lippman SA Gilmore HJ Lane T Radebe O Chen Y Mlotshwa N. Ability to use oral fluid and fingerstick HIV self-testing (HIVST) among South African MSM. PLoS ONE. (2018) 13:e0206849. 10.1371/journal.pone.0206849 30408055 PMC6224086

[73] Lippman SA Lane T Rabede O Gilmore H Chen Y Mlotshwa N. High acceptability and increased HIV testing frequency following introduction of HIV self-testing and network distribution among South African MSM. JAIDS. (2018) 77:279–87. 10.1097/QAI.0000000000001601 29210826 PMC5807184

[74] Marwa T Karanja S OSero J Orago A. The effects of HIV self-testing kits in increasing uptake of male partner testing among pregnant women attending antenatal clinics in Kenya: a randomized controlled trial. PAMJ. (2019) 33:213. 10.11604/pamj.2019.33.213.14160 31692660 PMC6814341

[75] Moore HA Metcalf CA Cassidy T Hacking D Shroufi A Steele SJ. Investigating the addition of oral HIV self-tests among populations with high testing coverage – do they add value? Lessons from a study in Khayelitsha, South Africa. PLoS ONE. (2019) 14:e0215454. 10.1371/journal.pone.0215454 31048859 PMC6497254

[76] Mugo PM Micheni M Shangala J Hussein MH Graham SM Rinke de Wit TF. Uptake and acceptability of oral HIV self-testing among community pharmacy clients in Kenya: a feasibility study. PLoS ONE. (2017) 12:e0170868. 10.1371/journal.pone.0170868 28125699 PMC5268447

[77] Pintye J Drake AL Begnel E Kinuthia J Abuna F Lagat H. Acceptability and outcomes of distributing HIV self-tests for male partner testing in Kenyan maternal child health and family planning clinics. AIDS. (2019) 33:1369–78. 10.1097/QAD.0000000000002211 30932954 PMC6546533

[78] Schaffer EM Gonzalez JM Wheeler SB Kwarisiima D Chamie G Thirumurthy H. Promoting HIV testing by men: a discrete choice experiment to elicit preferences and predict uptake of community-based testing in Uganda. Applied Health Econ Health Policy. (2020) 18:413–32. 10.1007/s40258-019-00549-5 31981135 PMC7255957

[79] Strauss M George G Landsell E Mantrell JE Govender K Romo ML. HIV testing preferences among long distance truck drivers in Kenya: a discrete choice experiment. AIDS Care. (2018) 30:72–80. 10.1080/09540121.2017.1367086 28847156 PMC5903847

[80] Strauss M George G Landsell E Mantrell JE Romo ML Mwai E. Stated and revealed preferences for HIV testing: can oral self-testing help to increase uptake amongst truck drivers in Kenya? BMC Public Health. (2018) 18:1231. 10.1186/s12889-018-6122-1 30400898 PMC6219162

[81] Thirumurthy H Masters SH Mavedzenge SN Maman S Omanga E Agot K. Promoting male partner testing and safer sexual decision making through secondary distribution of HIV self-tests by HIV uninfected female sex workers and women receiving antenatal and postpartum care in Kenya: a cohort study. Lancet HIV. (2016) 3:e266–74. 10.1016/S2352-3018(16)00041-2 27240789 PMC5488644

[82] Sambakunsi R Kumwenda M Choko A Corbett EL Desmond NA. ‘Whose failure counts?' A critical reflection on definitions of failure for community health volunteers providing HIV self-testing in a community-based HIV/TB intervention study in urban Malawi. Anthropol Med. (2015) 22:234–49. 10.1080/13648470.2015.1077202 26762610 PMC4720041

[83] Neuman M Indravudh P Chilongosi R d'Elbee M Desmond N Fielding K. The effectiveness and cost-effectiveness of community-based lay distribution of HIV self-tests in increasing uptake of HIV testing among adults in rural Malawi and rural and peri-urban Zambia: protocol for STAR (self-testing for Africa) cluster randomize evaluations. BMC Public Health. (2018) 18:1234. 10.1186/s12889-018-6120-3 30400959 PMC6218995

[84] Deville W Tempelman H. Feasibility and robustness of an oral HIV self-test in a rural community in South-Africa: an observational diagnostic study. PLoS ONE. (2019) 14:e0215353. 10.1371/journal.pone.0215353 30986228 PMC6464222

[85] Simwinga M Kumwenda MK Dacombe RJ Kayira L Muzumara A Johnson CC. Ability to understand and correctly follow HIV self-test kit instructions for use: applying the cognitive interview technique in Malawi and Zambia. J Int AIDS Soc. (2019) 22:e25253. 10.1002/jia2.25253 30907496 PMC6432102

[86] Chipungu J Bosomprah S Zanolini A Thimurthy H Chilengi R Sharma A. Understanding linkage to care with HIV self-test approach in Lusaka, Zambia–a mixed method approach. PLoS ONE. (2017) 12:e0187998. 10.1371/journal.pone.0187998 29149194 PMC5693414

[87] Tun W Vu L Dirisu O Sekoni A Shoyemi E Njab J. Uptake of HIV self-testing and linkage to treatment among men who have sex with men (MSM) in Nigeria: a pilot programme using key opinion leaders to reach MSM. J Int AIDS Soc. (2018) 21:e25124. 10.1002/jia2.25124 30033680 PMC6055125

[88] MacPherson P Lalloo D Webb EL Maheswaran H Choko AT Makombe SD. Effect of optional home initiation of HIV care following HIV self-testing on antiretroviral therapy initiation among adults in Malawi. JAMA. (2014) 312:372–9. 10.1001/jama.2014.6493 25038356 PMC4118051

